# Neuroplasticity in middle age: an ecologically valid approach

**DOI:** 10.3389/fnhum.2012.00324

**Published:** 2012-11-29

**Authors:** Rachael D. Seidler

**Affiliations:** School of Kinesiology and Department of Psychology, University of MichiganAnn Arbor, MI, USA

**A commentary on**

**The effect of leisure activity golf practice on motor imagery: an fMRI study in middle adulthood**

by Bezzola, L., Merillat, S., and Jancke, L. (2012). Front. Hum. Neurosci. 6:67. doi: 10.3389/fnhum.2012.00067

“*You know you're middle aged when caution is the only thing you care to exercise*.”

In “The effect of leisure activity golf practice on motor imagery: an fMRI study in middle adulthood,” Bezzola et al. (2012) demonstrate changes in the neural representations of imagined movement following 40 h of golf training. An experimental (golf novice) and control group were scanned using functional MRI during kinetic motor imagery of their golf swing both prior to and following the golf training (with the control group matched for average pre- to post-test duration). The authors found reductions in bilateral dorsal premotor cortex activity during motor imagery following the training period only in the experimental group and not the control group, suggesting more efficient neural representations following training.

This study is unique among the large number of papers that have been published in recent years on experience-dependent sensorimotor neuroplasticity. One defining feature of this work is that the characteristics of the training program were not regulated. The experimental group simply participated in golf training for a total of 40 h. This ecologically valid approach makes the findings generalizable to what individuals may choose to do for their own leisure and exercise on any given day. This indicates that precisely regimented training programs are not *de rigueur* for behavioral and brain plasticity to occur.

Secondly, the participants ranged in age from 40 to 60 years. Research on the cognitive neuroscience of aging has begun to yield insights into age differences in motor control and learning at both the behavioral and brain levels (cf. Seidler et al., 2010). However, work with individuals in the middle aged range of the lifespan is scant. It is important to investigate both behavioral and neural plasticity within this age range, however, because these individuals make up a large portion of the workforce. Moreover, such an approach will be critical for identifying trajectories of decline. The few studies that have investigated behavioral and brain function of participants in this age range have yielded the sobering finding that many changes evident in older adults are already manifest at this point. For example, individuals aged 40–60-years old exhibit evidence of declines in sensorimotor adaptation in comparison to those in their twenties (Heuer and Hegele, 2011). Interestingly, retention of the ability to transfer learning to new conditions is preserved (Heuer and Hegele, 2011), similar to what has also been reported in older individuals (Seidler, 2007; Langan and Seidler, 2011).

Likewise, numerous studies have shown that older adults tend to under-recruit task relevant brain regions while over-activating additional areas in comparison to young adults (Langan et al., 2010; Seidler et al., 2010). Different theories exist regarding the function of this over-activation (cf. Lindenberger et al., 2012), including the compensation viewpoint and the nonselective recruitment or dedifferentiation view. The compensation view posits that this over-recruitment serves to compensate for age-related brain structural and biochemical declines and is associated with better performance (cf. Reuter-Lorenz and Lustig, 2005). In contrast, dedifferentiation suggests that over-recruitment is a sign of less efficient or specific neural representations with age (Li and Lindenberger, 1999). Recent findings support the Compensation-Related Utilization of Neural Circuits Hypothesis (CRUNCH), which posits that older adults reach their limits of cognitive and neural resources at lower levels of task difficulty (Reuter-Lorenz and Cappell, 2008; Carp et al., 2010; Schneider-Garces et al., 2010). That is, they exhibit over-activation as a compensatory mechanism at lower levels of task difficulty, and once they have reached their maximum capacity then activation declines. Functional brain over-activation is already apparent in individuals that are 60-years old (Burgmans et al., 2010). Moreover, studies investigating brain volumetric changes with age indicate that losses are evident in those within the range of 40–60-years old, particularly for brain structures involved in learning and memory (Ziegler et al., 2012).

Thus, although behavioral changes may not be as marked in middle aged individuals as they are in older adults, brain structure and function are exhibiting evidence of age differences. United States Census data predict that there will be 88.5 million people in the US over the age of 65 by the year 2050. This dramatic shift in the population will increase the need for programs and interventions that can facilitate activities of daily living for older adults. Further investigation of neuroplastic changes within middle aged individuals will be important for providing prescriptions regarding the optimal time point for motor training interventions. Bezzola et al. (2012) have gotten us onto the fairway by providing evidence for neuroplastic changes in middle aged adults using a realistic and ecologically valid paradigm.
